# Characterisation of Fe Distribution in the Liquid–Solid Boundary of Al–Zn–Mg–Si Alloy Using Synchrotron X-ray Fluorescence Microscopy

**DOI:** 10.3390/ma17143583

**Published:** 2024-07-19

**Authors:** He Tian, Dongdong Qu, Nega Setargew, Daniel J. Parker, David J. Paterson, David StJohn, Kazuhiro Nogita

**Affiliations:** 1 School of Mechanical and Mining Engineering, The University of Queensland, St Lucia, QLD 4072, Australia; 2 Product Innovation and Technology, BlueScope Steel Australia, Port Kembla, NSW 2505, Australia; 3 Australian Synchrotron, ANSTO, Clayton, VIC 3168, Australia

**Keywords:** Al–Zn-based alloy, directional solidification, synchrotron X-ray fluorescence microscopy, intermetallics, Fe

## Abstract

Al–Zn–Mg–Si alloy coatings have been developed to inhibit the corrosion of cold-rolled steel sheets by offering galvanic and barrier protection to the substrate steel. It is known that Fe deposited from the steel strip modifies the microstructure of the alloy. We cast samples of Al–Zn–Mg–Si coating alloys containing 0.4 wt% Fe and directionally solidified them using a Bridgman furnace to quantify the effect of this Fe addition between 600 °C and 240 °C. By applying a temperature gradient, growth is encouraged, and by then quenching the sample in coolant, the microstructure may be frozen. These samples were analysed using scanning electron microscopy (SEM) and energy dispersive X-ray spectroscopy (EDS) to determine the morphological effects of the Fe distribution across the experimental temperature range. However, due to the sub 1 wt% concentration of Fe, synchrotron X-ray fluorescence microscopy (XFM) was applied to quantitatively confirm the Fe distribution. Directionally solidified samples were scanned at 7.05 keV and 18.5 keV using X-ray fluorescence at the Australian Synchrotron using the Maia array detector. It was found that a mass nucleation event of the Fe-based τ_6_ phase occurred at 495 °C following the nucleation of the primary α-Al phase as a result of a peritectic reaction with remaining liquid.

## 1. Introduction

The Al-43.4wt%Zn-2wt%Mg-1.6wt%Si (Al–Zn–Mg–Si) alloy is used as a corrosion-resistant coating for steel sheets and offers superior cut and barrier protection against corrosion [1]. The coated steel is produced on a continuous hot-dip galvanising line, where a steel sheet is run into the hot liquid alloy bath and is then pulled out after it turns around a sink roll immersed inside the bath. However, on the production line, Fe is constantly deposited into the liquid bath, leading to the formation of unwanted intermetallic compounds (IMCs), which must be manually removed from the bath [2]. These unwanted IMCs are treated as three types, which are top dross, suspended dross and bottom dross. Top dross located on the surface of the coating bath can be scraped off during operation. A small proportion of the dross remains suspended floating in the melt, and dross that settles at the bottom of the bath requires the coating bath to be removed and the dross manually removed. Neither the frequent cleaning of the hot dipping baths nor the potential inclusion of dross in the coated strip are practical, so an understanding of the dross formation mechanism is valuable [2,3]. Reduced bath cleaning frequency has economic benefits for the manufacturer, and a higher-quality coating will yield superior performance for the end user.

The degree of IMC formation has been determined to be a function of the Fe content in the coating bath, which has been found to reach equilibrium solubility at 0.4 wt% Fe in the Al–Zn–Mg–Si alloy bath at 600 °C [2]. During the manufacturing process, the nominal 600 °C bath is continuously topped up with fresh liquid Al–Zn–Mg–Si alloy. The virgin alloy added to the bath is known to contain a nominal 0.1 wt% Fe because of the alloy preparation process [2]. The two primary Fe-bearing phases of interest are the τ_5c_ Al_20_Fe_5_Si_2_ and the τ_6_ Al_9_Fe_2_Si_2_ phases, of which the crystal lattices are determined to be body-centred cubic (bcc) [4] and monoclinic [5], respectively. The bcc structure of the $\tau_{5}$ phase results from the presence of Zn in the alloy, which transforms the α phase. The τ_5c_ Al_20_Fe_5_Si_2_ (+Zn) equilibrium IMC phase usually forms on the substrate steel, below the coating alloy [6]. However, it also forms within the solution, where it can also act as a reservoir for Fe when bath temperatures rise [7]. The bcc structure of the τ_5c_ phase is the result of trace transition metals in the alloy, and in the case of the Al–Zn–Mg–Si alloy analysed, incidental Cr is believed to be the main stabilising agent [8]. The goal of this paper is to determine the distribution of Fe in the alloy system as it solidifies as well as to form a hypothesis on the formation mechanisms of IMCs. A challenge for understanding the formation mechanisms for these IMCs is that although the IMCs themselves contain enough Fe for conventional analysis techniques, the nominal concentration of Fe in the liquid phase and liquid–solid boundary is very low. Therefore, they often cannot be reliably detected. This paper aims to address this challenge. It should be noted that the main focus of this paper is the fundamentals of Fe-bearing IMC formation in this alloy system, and the performance of the coating alloy or the coated product will not be discussed.

Previous work by Tong et al. [9] found that the primary Fe-containing IMCs of interest consist of τ_6_ and τ_5c_, and the formation temperature as well as the phase fraction of τ_6_ have been found to increase with increasing Fe content. It was found in bulk air-cooled samples that with less than 0.4 wt% Fe, the τ_5c_ phase would fail to appear. Above the liquidus temperature of the alloy, the Fe is believed to stay suspended in solution; however, upon cooling below the liquidus temperature, these IMCs are known to nucleate and grow from the liquid and are distributed in the interdendritic region within the α-Al network afterwards. Thermal analysis by Tong et al. [9] identified the formation temperatures of several primary phases including α-Al and various Zn-/Mg-containing phases. However, due to the low concentration of Fe in the alloy mixture, the energy released in the solidification events is not enough to be observed. Observation using SEM EDS has identified that these phases nucleate at a similar temperature to the much more prominent α-Al phase, suggesting that their solidification peaks have overlapped with that of the α phase [9]. The solubility of Fe in the Al–Zn–Mg–Si alloy has been quantified by Setargew et al. [2] as

(1) $$\ln\left[Fe\right]=-\frac{7220}{T}+7.39,$$

**Figure 1:**
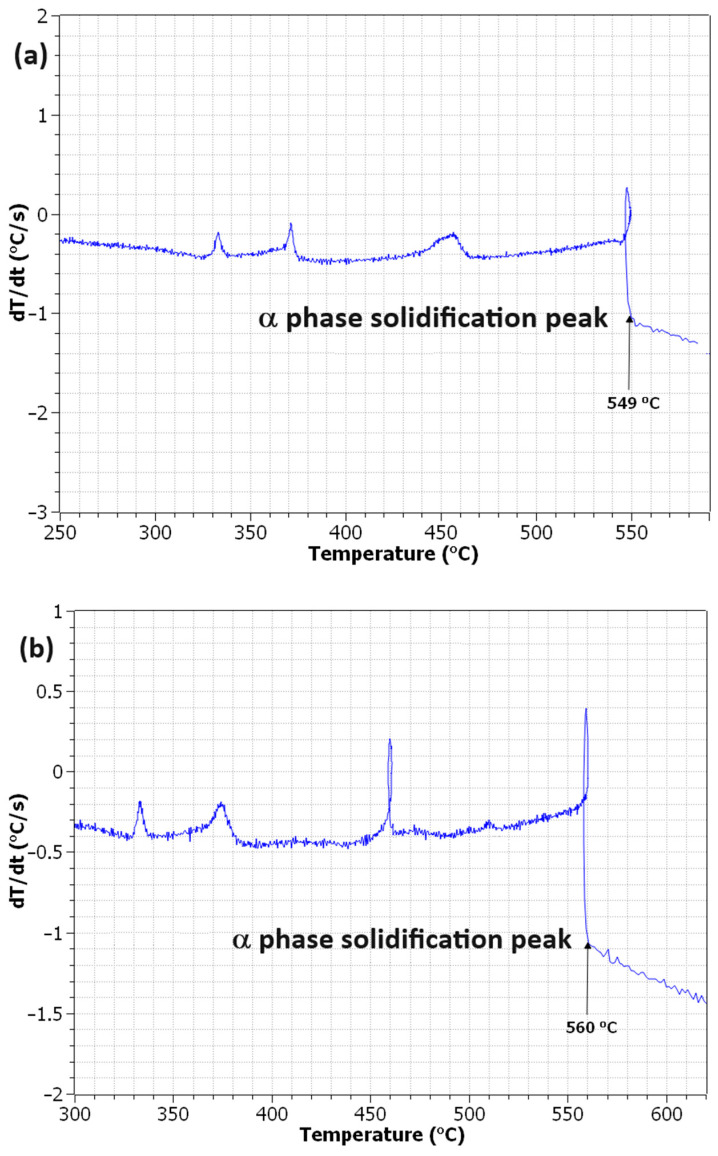
Cooling curve analyses for (**a**) 0.4 wt% Fe Al–Zn–Mg–Si alloy and (**b**) 0.1 wt% Fe Al–Zn–Mg–Si alloy.

Here, *T* is the absolute temperature of the coating bath. In this experimental series, the bath temperature is analogous to the temperature of the sample at the time of quench. As the sample undergoes rapid cooling during the quench, Fe is also rapidly segregated out of the melt. At 600 °C, the solubility of Fe in the melt is 0.41 wt%, but by the time it has cooled to 500 °C, the equilibrium concentration has decreased to 0.14 wt%. This is predicted to cause a high degree of constitutional undercooling ahead of the solidification front, creating favourable conditions for the nucleation of IMCs, which depends on the segregation of Fe out of the solution to form. It can be seen from the Al-rich end of the binary Al–Fe phase diagram [10] that up to the first eutectic point of approximately 2 wt%, adding Fe has a stabilising effect on the liquidus temperature of the system. Figure 1 shows a decrease in the formation temperature of the highest temperature formation peak, corresponding to the α phase [10] when Fe content is increased from 0.1 wt% to 0.4 wt%. When Fe content is increased from 0.1 wt% to 0.4 wt%, the formation temperature of the α phase decreases from 560 °C to 549 °C, showing the stabilising effect of the Fe. It is predicted that this lower formation temperature for the α phase will result in a lower formation barrier for Fe-based IMCs. Lastly, another driving factor for the formation of Fe-based IMCs is believed to be the presence of a thermal gradient, which was found to have a dominant effect on the formation of Fe-bearing IMCs compared to the cooling rate, as found by Feng et al. [11]. The presence of a thermal gradient during solidification as well as the alloy temperature can be seen as critical in the formation mechanism of IMCs in the Al–Zn–Mg–Si alloy. This paper will attempt to verify the mechanisms behind these observations.

## 2. Materials and Methods

### 2.1. Casting and Initial Preparation of Samples

**Figure 2:**
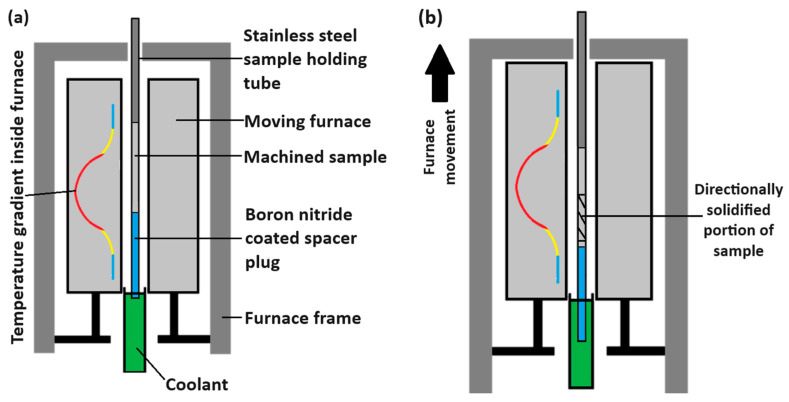
DS apparatus used for solidifying AM samples against a temperature gradient. (**a**) Shows a labelled schematic of the sample and furnace prior to a DS experiment, and (**b**) shows the positioning after. The cross-hatched portion of the sample in (**b**) has been re-melted and solidified inside the furnace against the temperature gradient and is the portion of interest.

Samples were prepared using an industrial Al–Zn–Mg–Si master alloy. The master alloy was re-melted in an induction furnace and cast into a graphite pencil mould. Then, the samples for the directional solidification (DS) tests were machined out of the cast pencil sample into a 4.5 mm diameter rod of approximately 100 mm length using a lathe. These rods were then inserted into a 1.6 mm wall-thickness 316L stainless-steel tube, which was attached to the frame of the Bridgman furnace, as seen in Figure 2.

### 2.2. Directional Solidification and Sample Mounting

A temperature gradient of 6.2 K/mm was applied to the DS sample and the sample was pulled for a total of 60 mm at a rate of 0.02 mm/min, then rapidly quenched in a refrigerated ethylene glycol-based coolant bath held at 2 °C. The samples were sectioned parallel to the direction of pull and mounted in 25 mm long segments in Struers Polyfast conductive resin, then ground and polished using silicon carbide sandpaper and Struers Diapro Nap B and Mol B solution until fit for SEM observation (JEOL 7800F (JEOL, Tokyo, Japan) at 15 keV accelerating voltage, probe current set to 11 (1000 pA), aperture size of 4, backscattered and with 10 mm working distance).

### 2.3. Description of Mounted Samples and Temperature Zones

The temperature range of the directionally solidified section was calculated to be between 240 °C and 600 °C by calibrating the furnace first using a Type N thermocouple. The three 25 mm sample segments, henceforth indicated as ‘low’, ‘medium’ and ‘high,’ were calculated to correspond to the following temperature ranges: 240–310 °C, 310–480 °C and 480–600 °C. These temperatures were calculated by referencing the temperature gradient recorded inside the furnace with the Type N thermocouple against the position of the sample inside.

### 2.4. XFM Sample Preparation

Samples for the synchrotron X-ray fluorescence (XFM) were prepared by using a diamond saw to slice a thin wafer off the resin-mounted samples; then, using a handheld wafer carrier, the films were ground down to nominal 30 μm thin films, verified using a micrometre. The synchrotron XFM analysis was performed at the XFM beamline at the Australian Synchrotron. The samples were scanned using an incident X-ray beam of 18.5 keV with the scanning pixel size 1 μm × 1 μm. The reflected X-ray signal was recorded using the Maia 385 array detector. In metallurgical studies, alternative techniques such as X-ray dispersive spectroscopy (EDS) and electron backscatter diffraction (EBSD) are used for phase identification and the analysis of crystallography, but this paper will attempt a novel application of XFM on a metallurgical sample instead. This is due to the higher sensitivity limits for detecting low concentration elements (<1 wt%), and for determining their distribution.

### 2.5. Phase Verification

The detailed verification and identification of the IMC phases found in this paper were out of scope, as the primary objective of the paper was to study the formation mechanism of these phases. Hence, only SEM-EDS was used to verify the composition of IMC crystals found in this study, which are already well known in the context of this alloy system. In-depth phase identification can be found in the works by Setargew et al., Qu et al. and Khaliq et al. [2,3,7].

### 2.6. Verification of Stainless-Steel Reaction

It should be noted that the sample studied using DS and subsequent XFM samples were generated by directly inserting the Al–Zn samples into an uncoated stainless-steel tube. A side effect of this direct insertion into the tube is the formation of an IMC phase between the wall of the tube and the alloy, resulting from the dissolution of mainly Fe, but also trace amounts of Cr and Ni, into the liquid Al–Zn alloy inside the tube to form the modified τ_5c_ phase, which occurs as a result of Cr and Zn substituting Fe atoms in the $\tau_{5c}$ phase [12,13]. This reaction may be seen in XFM images in the latter part of the paper.

An adverse effect of the dissolution of Fe into the liquid is that the nominal Fe content of the alloy can no longer be 0.4 wt%, with much higher local Fe concentrations suspected in the vicinity of the stainless-steel tube wall. To verify that this additional Fe dissolution has no or a negligible effect on the solidification sequence of the alloy, a literature review was performed, and EDS analysis of the Fe content in the boundary region was performed. Setargew [12] found that when a rectangular coupon made of 316L stainless steel was dipped into an alloy coating bath, two distinct IMC layers would form, with the first layer (directly bordering the stainless-steel substrate) being denoted as AL1 and the second AL2, with AL2 resulting in small clusters of IMCs that break off and drift into the liquid coating alloy. The composition of the AL2 layer found by Setargew [12] is seen in Table 1. This was compared to the composition of an IMC crystal observed in a directionally solidified sample, which was believed to result from direct contact between the stainless-steel tube and the alloy. The compositions are similar, except there is a considerably lower amount of Cr content in the directionally solidified samples, which can be attributed to limited diffusion time within the Bridgman furnace. Both the coupon used by Setargew and the tube in the directionally solidified sample were manufactured out of 316L stainless steel.

**Figure 3:**
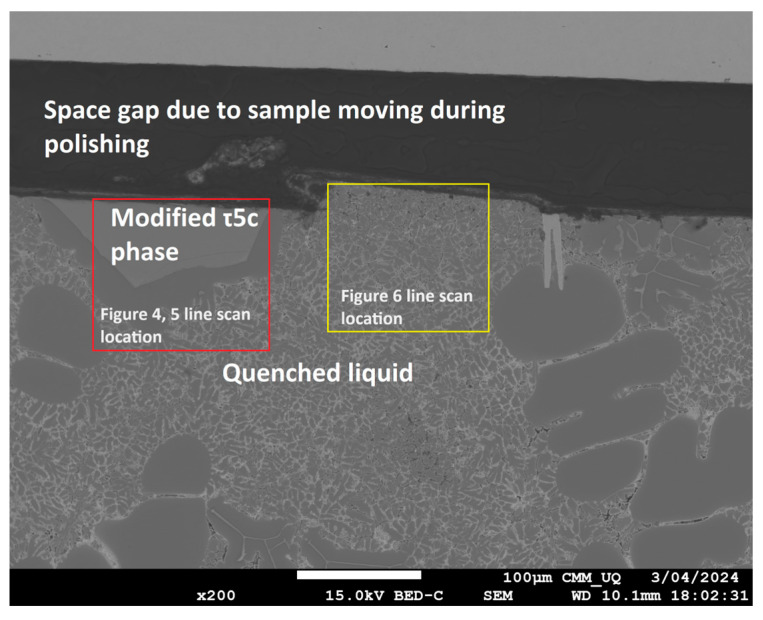
SEM EDS image taken using JEOL 7800 showing the presence of the modified τ_5c_ phase.

**Figure 4:**
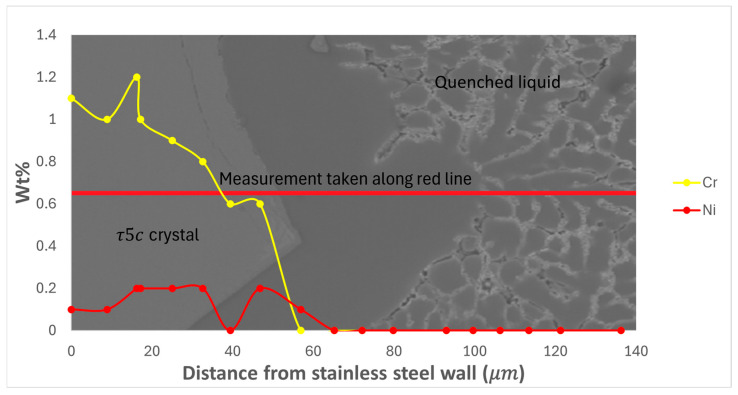
Wt% of Cr and Ni against distance from the stainless-steel tube wall in a sample where the modified τ_5c_ reaction has occurred.

**Figure 5:**
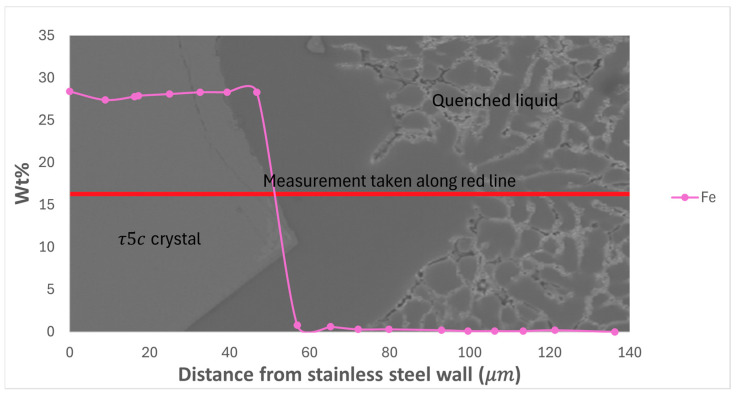
Wt% of Fe against distance from the stainless-steel tube wall in a sample where the modified τ_5c_ reaction has occurred.

**Figure 6:**
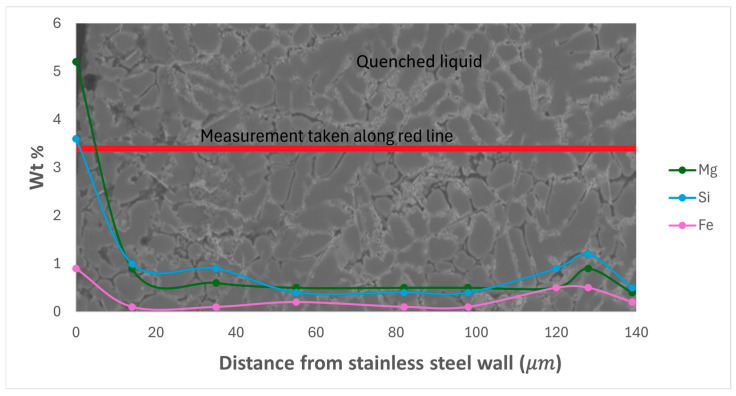
Wt% of Mg, Si, and Fe versus distance from stainless-steel tube wall in a sample where the modified τ_5c_ reaction has not occurred.

EDS measurement of two types of area in the ‘high’ part of the directionally solidified sample containing 0.4 wt% Fe was undertaken, with the two measurement areas consisting of a location where the modified τ_5c_ IMC has formed and the second consisting of a point where there has been direct contact between the stainless-steel tube and the liquid alloy but with no visible reaction, as illustrated in Figure 3. The composition of the alloy with distance from the stainless-steel tube was mapped and is presented in Figure 4, Figure 5 and Figure 6. The deposition of Cr and Ni from the stainless steel was exclusively limited to the modified τ_5c_ phase (verified with SEM-EDS), with no further dissolution into the quenched liquid, as seen in Figure 4. Figure 4 shows that outside the crystal of the modified τ_5c_ phase, the Cr and Ni content drops to undetectable levels. It is believed that this is due to Cr and Ni’s low solubility in the Al liquid. The composition of the liquid was found to stabilise quickly with increasing distance from the stainless-steel tube wall, with Fe content decreasing sharply following the transition from the modified τ_5c_ crystal to α-Al and the quenched liquid, as seen in Figure 5. In areas where there is no visible reaction between the stainless-steel tube and the alloy, the Fe content is seen to be elevated near the edge of the stainless-steel tube but quickly drops to equilibrium within 60 μm. Likewise, even in samples where the modified τ_5c_ phase has formed, the composition stabilises within approximately 20 μm, as seen in Figure 6.

Although the nominal Fe content could no longer be considered to be 0.4 wt% due to the extensive dissolution of Fe into the liquid, this reaction is largely contained within a thin reaction layer. The centre of the alloy sample is unaffected and can still be used to study the solidification mechanics of Fe-based IMCs.

## 3. Results and Discussion

### 3.1. Directional Solidification

**Figure 7:**
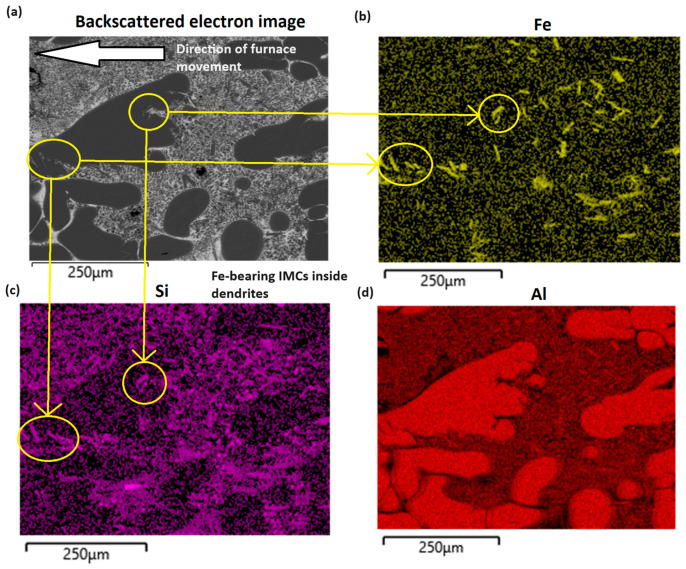
SEM EDS capture showing the composition of a directionally solidified Al–Zn–Mg–Si sample. (**a**) Backscattered electron image (**b**) Fe content (**c**) Si content (**d**) Al content.

Prior to investigation using XFM, SEM-EDS analysis was employed to investigate the Fe distribution in the alloy. The primary areas of interest lay within the boundary between the quenched liquid and the site of early primary α-Al dendrite formation, as this is where Fe-based IMCs were predicted to nucleate. An image taken with a JEOL 7800F SEM of a nominal 0.4 wt% Fe-containing Al–Zn–Mg–Si DS sample (Figure 7) shows that at the liquid–solid boundary (LSB), needle-like crystals of the τ_6_ IMC phase are observed, with some of them embedded within α-Al dendrites. SEM-EDS spot analysis is used to verify their composition. SEM-EDS mapping shows the concentration of Fe to be largely concentrated within these IMCs (Figure 7). This solidification event of $\tau_{6}$ was calculated to occur at 495 °C by referencing the location of the event to the measured temperature gradient inside the furnace.

**Figure 8:**
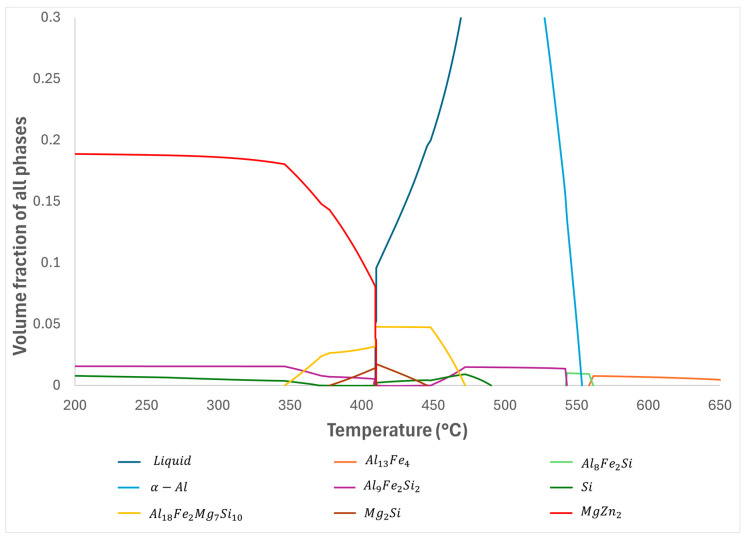
Thermo Calc one-axis equivalent phase diagram for 0.4 wt% Fe-containing Al–Zn–Mg–Si alloy [9].

While there is a possibility that these Fe-bearing IMC crystals grew because of the rapid coolant quench, their size and distribution indicate that they were already present at the time of quench. The IMCs nucleated in close succession to the α-Al phase, as predicted by the one-axis equivalent phase diagram in Figure 8. This is shown by the presence of IMC crystals embedded inside dendrites of the α-Al phase. SEM-EDS analysis of the α-Al phase composition along the temperature gradient found no clear trend in the concentration of Fe with decreasing temperature. This suggests that although Fe has a stabilising effect on the liquidus temperature of the α-Al phase, Fe only has limited solubility in the α-Al dendrites and remains mostly within the interdendritic liquid. This is verified using synchrotron XFM in the following section of this paper. It was noted that the highest concentrations of dissolved Fe occurred in the higher-temperature regions of the directionally solidified sample, consistent with Setargew et al.’s prediction [2] of decreasing equilibrium concentration with decreasing temperature, leading to the segregation of Fe out of solution.

The τ_6_ phase was predicted by the Thermo-Calc one-axis equivalent phase diagram, shown in Figure 8, to occur below 550 °C at a 0.4 wt% Fe concentration. Likewise, the τ_5_ phase is predicted to occur prior to the initial nucleation of the α-Al phase, and according to Gorny et al. [14], either undergoes a peritectic transformation into the τ_6_ phase or remains suspended in solution.

**Figure 9:**
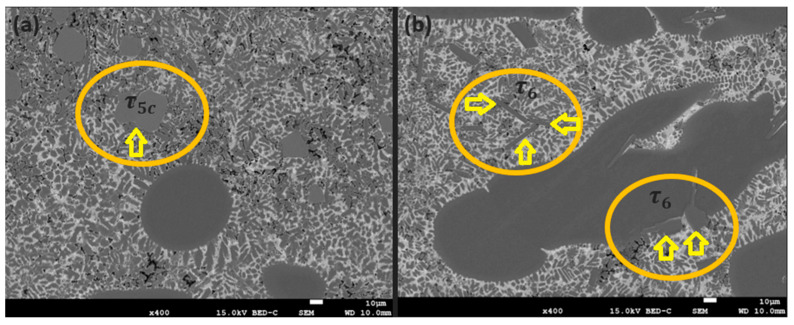
Backscattered SEM images of 0.4 wt% Fe-containing Al–Zn–Mg–Si alloy in two regions of the high-temperature zone showing (**a**) $\tau_{5c}$ and (**b**) $\tau_{6}$.

Crystals of the τ_5c_ phase were observed in the high-temperature (480–600 °C) region of the sample amidst the recently solidified α-Al phase, as seen in Figure 9. The $\tau_{5c}$ phase was observed in the absence of the similar τ_6_ phase, confirming that its formation preceded that of both the α-Al phase and the τ_6_ phase. The cubic τ_5c_ phase was largely observed to diffuse through the solidified microstructure via the interdendritic liquid, like the τ_6_ phase.

Although the τ_6_ phase typically has the appearance of a needle-like morphology, there have been instances where it has been observed with a broader-faceted morphology, not unlike the morphology of the divorced τ_5c_ crystals. This is taken as evidence that in three-dimensional space, the τ_6_ phase has a flake-like morphology. In Figure 9b, a perpendicular interaction of two τ_6_ flakes, verified by SEM-EDS, is observed, supporting this observation.

Although the τ_5c_ phase was observed in a Chinese script morphology in Tong et al.’s [9] work, it was only observed in a faceted divorced microstructure. It was shown by Awano and Shimizu [15] that the morphology of the resultant Fe-based IMCs can be dependent on the degree of superheat applied to Al–Si alloys before cooling, with increasing superheat associated with a higher abundance of Chinese script-like morphologies. A 750 °C superheating temperature was used by Tong et al. [9], resulting in a corresponding abundance of faceted/needle-like and Chinese-script morphologies. All Fe-based IMCs found in the directionally solidified sample analysed contained a mixture of needle-like or faceted microstructures with no Chinese script, indicating that the level of superheating was low.

For directionally solidified samples, the resultant microstructure of the final structure is heavily dependent on the ratio of the temperature gradient applied (*G*) to the growth rate (*V*), which is approximated as the rate of furnace movement. This ratio (*G*/*V*) can also be used to determine the transition point for constitutional undercooling at the LSB, using the expression provided by Stefanescu [16].

(2) $$\frac{G}{V}\geq \frac{m_{L}C_{0}\left(1-k\right)}{D_{L}},$$

The temperature gradient of −6150 K/m and pull rate of 8.33 × $10^{-6}$ m/s results in a *G*/*V* ratio of −7.38 × 10^8^ Ks/m^2^. The precise calculation of the right-hand-side of the equation in a quaternary alloy such as Al–Zn–Mg–Si is out of the scope for this paper—hence, approximations may be used instead by looking at the range of possible values for the concentration C_0_, diffusivity D_L_, liquidus slope m_L_ and partition coefficient k for the primary constituent binary system, namely Al–Zn. The value for the right-hand-side of the equation was calculated using the values for the Al–Zn binary system in Table 2 and was found to be equal to −5.372 × 10^4^ Ks/m^2^.

The difference in magnitudes of the left-hand-side and right-hand-side of the equation suggests that there is a high energy barrier for crystal growth. This corresponds to the high degree of segregation seen in solvents, including Fe, from the liquid phase during cooling, suggesting that the Al–Zn–Mg–Si alloy has high sensitivity to the cooling rate and Fe content. Gravity is another aspect that should be taken into consideration for this variety of DS experiment, as the sample is held vertically without stirring for an extended period. However, the nucleation of the τ_5c_ phase from the wall of the tube into the centre of the sample has been observed to be slow enough that diffusion of IMCs due to gravity was not an issue in the scope of this paper. From a practical perspective, if Fe content in the alloy cannot be controlled due to continued deposition from the steel sheet, some formation of these IMCs will be inevitable.

### 3.2. Synchrotron XFM

Although the presence and solidification order of the τ_5c_ and τ_6_ Fe-containing IMCs have been confirmed via prior studies, further work on the distribution of Fe in the liquid phase can still be undertaken.

**Figure 10:**
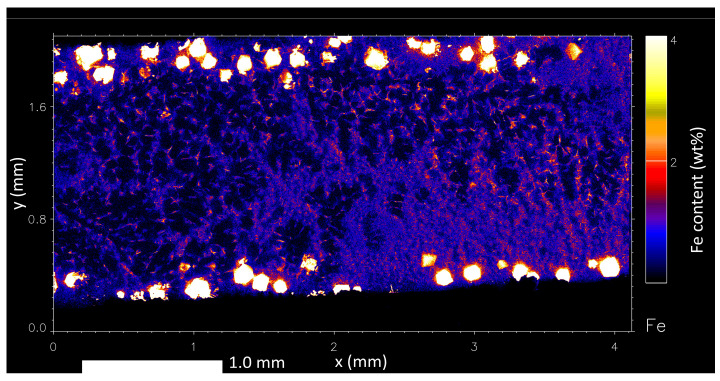
Full image of Fe distribution in the liquid–solid boundary in a nominal 0.4 wt% Fe-containing Al–Zn–Mg–Si alloy.

**Figure 11:**
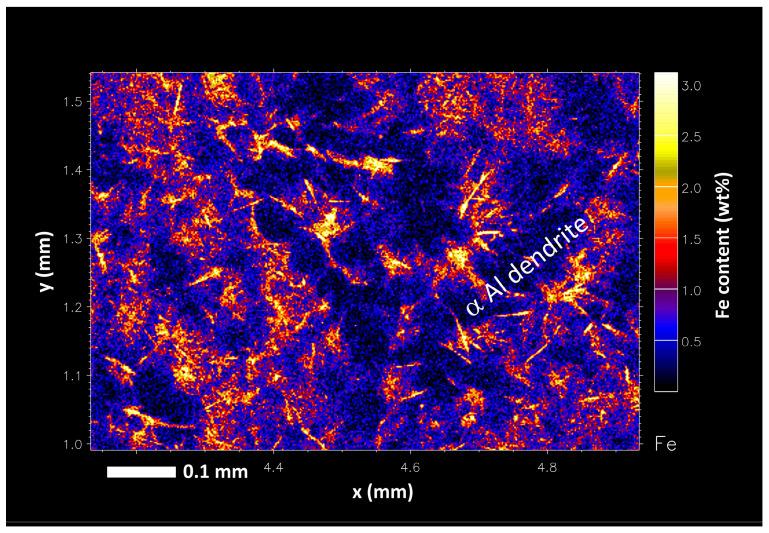
Cropped image of interdendritic Fe concentration in the liquid–solid boundary of a nominal 0.4 wt% Fe-containing Al–Zn–Mg–Si alloy.

Using synchrotron XFM, the distribution of Fe can be measured in directionally solidified samples to observe their distribution. In Figure 10, the concentration of Fe in the liquid at the LSB is observed to be consistent; however, much higher concentrations of Fe can be observed between primary α-Al phase dendrite arms, as seen in Figure 11. It is believed that as the α-Al dendrites solidify from the liquid, Fe is segregated into the liquid and becomes concentrated in areas of small dendrite arm spaces. This is supported by the positive concentration gradient for Fe radially from the centre of each α-Al dendrite, observed in Figure 11. It is through this that Fe concentrations reach high enough levels for the τ_6_ IMC to form. A high cooling rate applied to the liquid phase during the quench may result in the microsegregation of Fe out of solution to form the fine τ_6_ needles of less than 50 μm in length. The proposed IMC formation mechanism due to the microsegregation of Fe during cooling is supported by Gorny et al. and Christian [14,19], who suggested that Al–Fe–Si-based IMCs in the Al–Fe–Si systems often nucleate on the primary α-Al phase due to their favourable lattice matching. The presence of these fine τ_6_ particles shows that the τ_5_ phase formed from the precipitated Fe can bypass the reaction with trace transition metals to form τ_6_ instead of τ_5c_ by rapidly being cooled. It is thought that the presence of an α-Al dendrite network helps to raise the local concentration of Fe to encourage the formation of the τ_5_ phase. Although the level of Zn in these interdendritic pockets is also higher, it is believed that the formation of an τ_5c_ (Zn) IMC is bypassed during solidification, as suggested by Gorny et al. [14].

The peritectic reaction between the τ_5_ phase and liquid could only be valid for the formation of the τ_6_ phase as the τ_5c_ phase is known to form before a network of α-Al phases is grown from the liquid. This suggests that the τ_5c_ phase can also nucleate heterogeneously from an Fe-rich environment—in the case of the experimental samples, this surface is the wall of the stainless-steel sample tube, or Fe-rich liquid. Reduced τ_5c_ formation was observed between the wall and the alloy in samples where the inside of the tube had been coated using boron nitride, supporting this theory. It should be noted, however, that Tong [9] found τ_5c_ in bulk samples despite a lack of an Fe-rich interface to nucleate on. It should be established that the $\tau_{5c}\;$ particles that form between the wall and the alloy have a different morphology and composition to that which forms between α-Al dendrites. The phase that forms against the wall of the tube is τ_5c_ Al_8_Fe_2_Si (Cr), whereas the phase that forms in the interdendritic area is τ_5c_ Al_8_Fe_2_Si (Zn). Gorny et al. [14] noted that the cooling rate as well as Fe content had a strong influence on the IMCs formed in Al–Si alloys, with higher cooling rates increasing the likelihood of the transformation from τ_5_ to τ_6_. This partially agrees with the high cooling rate induced by the quench in refrigerated coolant resulting in a microstructure containing a mixture of both τ_6_ and τ_5c_. It is likely that the evolution from τ_5_ to τ_5c_ is responsible for its continued existence below the minimum temperature predicted by Figure 8, as the extra stability granted by its cubic structure has prevented it from participating in the peritectic reaction with the liquid to form more τ_6_. EDS analysis has been unable to detect the presence of the $\tau_{5}$ phase without modification, suggesting that the transformation from $\tau_{5}$ to $\tau_{5c}$ occurs in most incidences.

**Figure 12:**
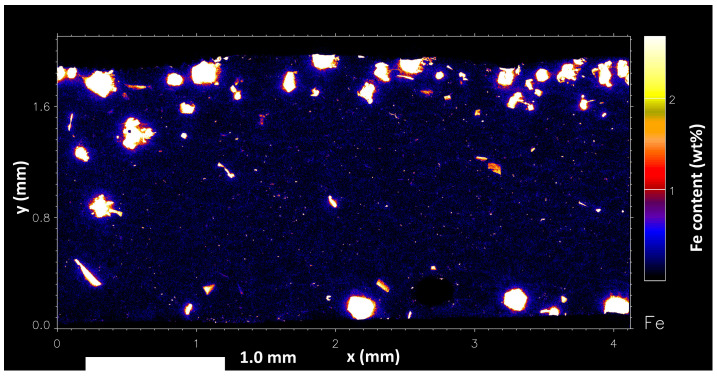
Full image of Fe distribution in the fully solidified (low temperature) region in a nominal 0.4 wt% Fe-containing Al–Zn–Mg–Si alloy.

In the lower-temperature region seen in Figure 12, the τ_6_ phase has been found to intersect with fully formed α-Al dendrites, confirming that it forms in close succession with the α-Al phase as predicted by the cooling curve analysis. Finer τ_6_ needles of approximately 50 μm in length can still be observed in the interdendritic liquid, resulting from the rapid quenching of the remaining liquid. The distribution of Fe becomes almost fully concentrated within the IMCs and only low concentrations are found in the interdendritic liquid, consistent with the exponential solubility relationship derived by [2]. This supports that Fe may have a stabilising effect on the Al-rich region of the Al–Fe binary system [10], with increasing Fe content causing the α-Al phase to nucleate at a lower temperature. There is a broader distribution of Fe in the liquid, where it is believed to help suppress the formation of α-Al. However, the concentration of Fe detected in the liquid region of the sample is shown to be up to 3 wt%, far exceeding the equilibrium concentration. This can be attributed to the rapid formation of IMCs during the quenching process, quantitative EDS of the liquid region has found that the average Fe concentration remains close to the equilibrium value. Average SEM-EDS mapping of the liquid (excluding any IMCs) has found the Fe concentration to remain close to or below the nominal 0.4 wt%, supporting the gradual segregation of Fe out of the liquid phase where it goes on to react and form IMCs.

In the lower-temperature region, it can be observed that the τ_6_ phase maintains good stability and persists with decreasing temperature. Fe solubility has decreased to the point that very little is still present in the interdendritic liquid, and most of the Fe is now contained in IMCs.

**Figure 13:**
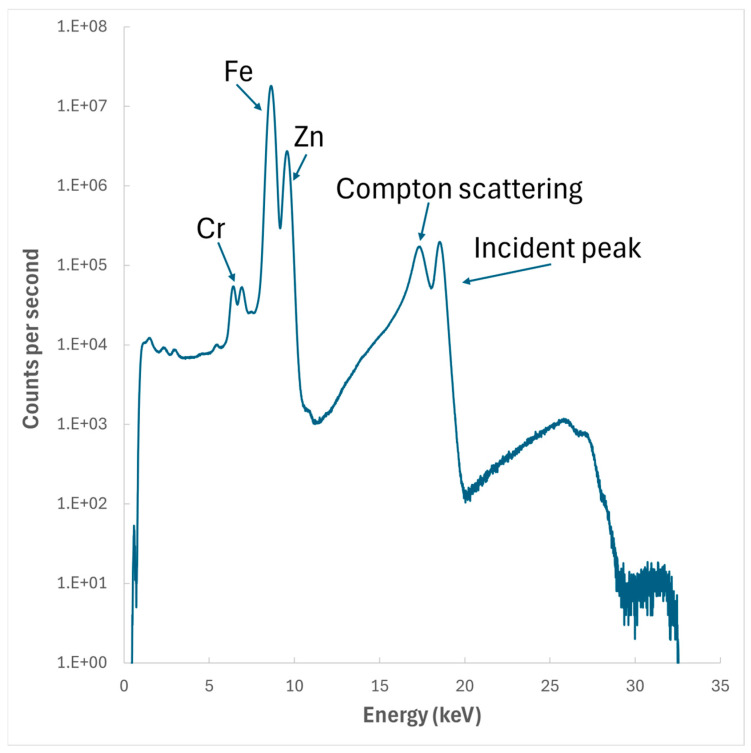
XFM spectra for nominal 0.4 wt% Fe-containing Al–Zn–Mg–Si sample.

The XFM spectra for the nominal 0.4 wt% Fe-containing Al–Zn–Mg–Si sample in Figure 13 shows strong peaks for Fe and Zn, as well as a peak for Cr that originates from the stainless-steel tube wall. The prevalence of this τ_5c_ (Cr) throughout the temperature range shows that by evolving to this cubic structure, it has been prevented from being transformed into the τ_6_ phase. The implications of this are that the formation of the τ_5c_ phase is sensitive to trace transition-metal additions in the coating bath, which are unavoidable. Without the formation of the τ_5c_ phase, τ_5_ would be free to undergo the peritectic reaction to form τ_6_. However, it could be argued that the concentration of the τ_5c_ phase along the edges of the stainless-steel tube is related to the faster cooling rate experienced by the outside of the sample.

**Figure 14:**
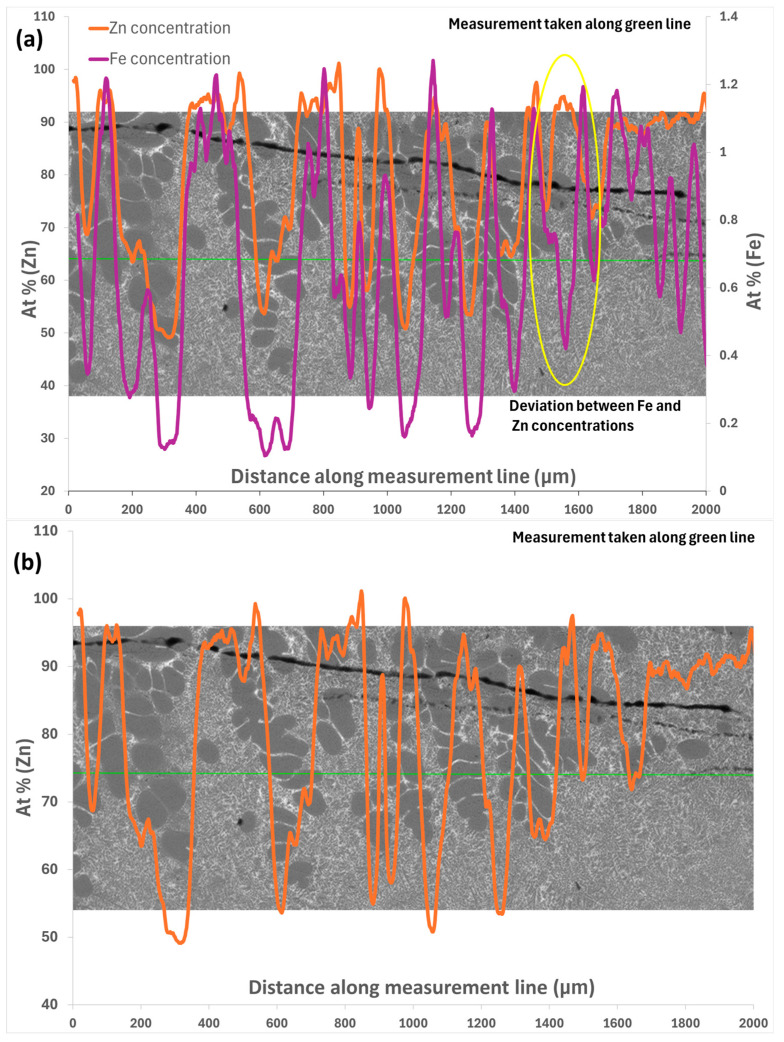

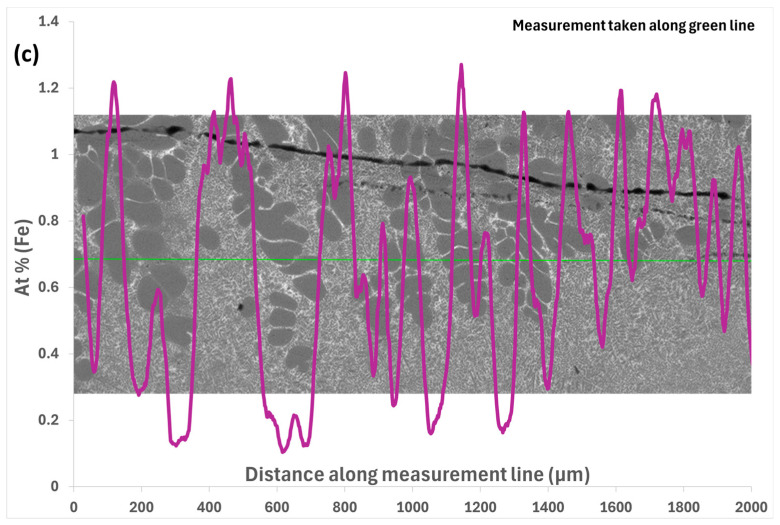
XFM line scan for nominal 0.4 wt% Fe-containing Al–Zn–Mg–Si sample in LSB. (**a**) Shows the deviation between the Fe and Zn concentration along the measurement line. (**b**) Shows only the concentration of Zn, and (**c**) shows only the concentration of Fe.

A scan was taken of the composition of the DS sample used in the XFM experiments to study the quantitative distribution of Fe in the LSB in Figure 14. The first finding of note was that the quantity of Fe increased significantly over areas of liquid compared to the solid α-Al phase, and the Fe content was generally found to increase in the direction of dendrite growth. Measurements were also taken in only the liquid region along the direction of growth, and it was found that the average Fe content in the liquid was approximately 0.90 at. %. Although this was still higher than that interior of α-Al dendrites, the Fe content of the liquid phase immediately adjoining an α-Al dendrite was found to be 1.00 at. % and decreased with the distance from the dendrite to the equilibrium concentration found in the liquid. This suggests that even in these early stages of solidification, there is a high degree of partitioning of Fe out from the solid phase. The line scan taken along the direction of growth traversing several α-Al dendrites and the interdendritic liquid pockets between them found that the Fe content between the dendrites increased to as much as 1.60 at. %. This suggests that the conditions the Fe-bearing-phase IMC required to nucleate coincide with a high degree of partitioning, and although the homogenous nucleation of Fe-bearing IMCs in the liquid, such as the smaller $\tau_{6}$ phase growths observed in the quenched interdendritic liquid, has been observed, nucleation is far more likely when Fe concentrations are highest between dendrite arms.

In Figure 14, the trend of both Fe and Zn content falling across α phase dendrites can be observed, with a sharp rise across areas of quenched liquid. The concentration is lowest at the middle of the dendrite due to interface layer effects and the active rejection of solutes during solidification. Although the concentrations of Zn and Fe differ greatly in magnitude, the peaks and valleys of the line scan in Figure 14 still show that they share the same trends across α-phase dendrites and the quenched liquid. There is an exception circled in Figure 14; however, analysis of the backscattered SEM image indicates that the discrepancy is most likely caused by a small Fe-bearing IMC particle in the liquid. An interesting observation is that from the LHS (cooler end) to the RHS (hotter end) of the graph, the average concentration of both Fe and Zn can be observed to increase across the positive temperature gradient. This is contrary to the Al–Fe and Al–Zn phase diagrams, which predict the solubility of Fe and Zn in Al to decrease with temperature [4]. It is possible then that the concentration gradient observed is a result of constitutional undercooling, which is known to create favourable conditions for the nucleation of new phases, especially Fe-bearing IMCs. The exception to this trend, observed in Figure 14, is at the transition from the final α-phase dendrite to the liquid: although the Zn concentration continues to increase according to the measured trend, the Fe concentration decreases sharply in the quenched liquid beyond the solidified dendrite network. This may be explained either by an error in measurement due to the relatively low concentration of Fe in the alloy, or by reviewing the Al-rich corner of the Al–Fe binary phase diagram [4], which shows the solubility of Fe decreasing with increasing temperature below 1.7 wt% Fe, due to the existence of a eutectic point. Hence, it can be speculated that during the cooling of the alloy, initially, the Fe concentration in the liquid decreases, but following this eutectic point, it will increase again to concentrations that may permit the homogenous nucleation of fine Fe-bearing IMC phases.

It is known that the $\tau_{5c}$ phase contains 31.91 at. % Fe [4] and the $\tau_{6}$ phase contains 27.20 at. % Fe [5], which is considerably greater than the nominal Fe concentration of 0.4 wt%. Analysis of the XFM spectra indicates that in both high- and medium-temperature regions, corresponding to the theoretical respective nucleation temperature ranges of these two phases, there are no pockets of interdendritic liquid showing concentrations of Fe equal to or higher than the atomic mass of Fe in the Fe-bearing intermetallics. It can therefore be speculated that the heterogenous nucleation of these IMCs is largely necessary, and that even with the maximum equilibrium concentration of bath Fe, homogenous nucleation is difficult. The exception to this is the formation of fine $\tau_{6}$ in the quenched liquid, but this may be explained by the rapid microsegregation of Fe within the fine α− Al dendrite network of the quenched liquid.

The right half of Figure 14 also shows that as the measurement area moves away from the dendritic network, the Fe content drops significantly with the distance from the α Al dendrites, supporting the notion that the dendritic network helps to concentrate Fe and that the average concentration of Fe in the liquid is low compared to the space between dendrites.

Areas in measurement where the concentration does not seem to correspond to the microstructure are believed to be a result of IMC inclusions, as the microstructure is not entirely homogeneous.

## 4. Conclusions

By using directional solidification to generate samples of the Al–Zn–Mg–Si alloy and analysing them using SEM EDS and synchrotron XFM, it was discovered that

1. As the α-Al phase solidifies, it partitions solutes such as Fe and Zn into the interdendritic regions, where concentrations can reach sufficient levels to enable the formation of IMCs.
2. Fe-bearing IMCs require conditions with a high concentration of Fe to form—in this case, because of segregation during cooling. $\tau_{6}$ forms in close succession to α-Al because the dendrite network forms the Fe-rich area required.
3. The $\tau_{6}$ phase has been shown to solidify in short succession to the α- Al phase, likely in a peritectic reaction between the $\tau_{5}$ phase and partitioned Zn from the interdendritic liquid at approximately 495 °C.
4. Although there is a high degree of partitioning of Fe into the liquid, it is still below the level of Fe contained in the IMCs themselves, so it is suspected that heterogenous nucleation is required for the formation of any Fe-based IMC.

## Figures and Tables

**Table 1:** SEM/EDS analyses of IMCs formed because of interaction between stainless steel and Al–Zn–Mg–Si coating alloy.

**Alloy Layer**	**Al**	**Cr**	**Fe**	**Ni**	**Zn**	**Si**
AL2 [12]	57.2 ^1^	6.70	25.35	0	3.56	7.20
Crystal identified in DS sample	56.90	1.10	28.4	0.10	6.60	6.60

^1^ All values given in wt.%.

**Table 2:** Concentration, diffusivity, liquidus slope and partition coefficient for Zn in an Al–Zn alloy.

Variable	Value	Units	References
$C_{0}$ (concentration)	41.1	wt%	−
$D_{L}$ (diffusivity)	1200	${\mu m}^{2}s^{-1}$	[17]
$m_{L}$ (liquidus slope)	−2.93	$K{\left(\mathrm{wt}\%\right)}^{-1}$	[17,18]
k (partition coefficient)	0.45	−	[17]

## Data Availability

The original contributions presented in the study are included in the article, further inquiries can be directed to the corresponding author.

## References

[1] BlueScope Activate Technology. http://www.steel.com.au/articles/article-46-activate-technology.

[2] Setargew N., Hodges J., Parker D. (2015). Dross Intermetallic Compound Formation and the Alloy Layer in 55%Al-Zn Coating. Proceedings of the Steel Research Hub Symposium, University of Wollongong.

[3] Wang L., Wang Z., Zhang S., Wei K., Guo Z. (2022). Recovery of aluminum–zinc alloy from 55%Al–Zn dross by supergravity separation. Rev. Sci. Instrum..

[4] Phragmen G. (1950). On the Phases Occurring in Alloys of Aluminium with Copper, Magnesium, Manganese, Iron, and Silicon. J. Inst. Met..

[5] Hansen V., Hauback B., Sundberg M., Rømming C., Gjønnes J. (1998). β-Al4.5FeSi: A Combined Synchrotron Powder Diffraction, Electron Diffraction, High-Resolution Electron Microscopy and Single-Crystal X-ray Diffraction Study of a Faulted Structure. Acta Cryst. B.

[6] Cheng W.-J., Wang C.-J. (2011). Effect of silicon on the formation of intermetallic phases in aluminide coating on mild steel. Intermetallics.

[7] Khaliq A., Kasva V., Alghamdi A.S., Ramadan M., Subhani T., Haider W., Abdel Halim K.S. (2024). Iron Intermetallic Compounds (IMCs) Formation Mechanism in the Molten Aluminium Zinc (Al-Zn) Coating Alloy. Teh. Vjesn..

[8] Cheng W.-J., Wang C.-J. (2011). EBSD study of crystallographic identification of Fe–Al–Si intermetallic phases in Al–Si coating on Cr–Mo steel. Appl. Surf. Sci..

[9] Tong Z. (2021). Solidification and Microstructural Characterization of Zn-Al Based Coating Alloys Research.

[10] Taylor J.A. (2012). Iron-containing intermetallic phases in Al-Si based casting alloys. Procedia Mater. Sci..

[11] Feng S., Liotti E., Lui A., Wilson M.D., Connolley T., Mathiesen R.H., Grant P.S. (2020). In-situ X-ray radiography of primary Fe-rich intermetallic compound formation. Acta Mater..

[12] Setargew N., Yuen W.Y.D., Hodges J.C. (2016). Intermetallic spike growth mechanisms in 316L stainless steel in contact with molten 55%Al-Zn metal coating alloy. Metall. Res. Technol..

[13] Cheng W.-J., Wang C.-J. (2013). Effect of chromium on the formation of intermetallic phases in hot-dipped aluminide Cr–Mo steels. Appl. Surf. Sci..

[14] Gorny A., Manickaraj J., Cai Z., Shankar S. (2013). Evolution of Fe based intermetallic phases in Al–Si hypoeutectic casting alloys: Influence of the Si and Fe concentrations, and solidification rate. J. Alloys Compd..

[15] Awano Y., Shimizu Y. (1987). Crystallization of AlFeSi Compound in Chinese Script Form in Al-Si Alloy Castings Melt-Superheated. J. Jpn. Foundrymen’s Soc..

[16] Stefanescu D.M. (2009). Science and Engineering of Casting Solidification.

[17] Liu P., Dunlop G.L. (1988). Crystallographic orientation relationships for Al-Fe and Al-Fe-Si precipitates in aluminium. Acta Metall..

[18] Acer E., Çadırlı E., Erol H., Kaya H., Gündüz M. (2017). Effects of Growth Rates and Compositions on Dendrite Arm Spacings in Directionally Solidified Al-Zn Alloys. Metall. Mater. Trans. A Phys. Metall. Mater. Sci..

[19] Christian J. (1959). Constitution of binary alloys (Second edition), by M. Hansen, with the cooperation of K. Anderko. Pp. xix + 1305. McGraw-Hill Book Co. Inc., New York; McGraw-Hill Publishing Co. Ltd, London. 1958. E12 12s. net. Endeav. (New Ser.).

